# Antibiotics prescribing practice among patients with urinary tract infection at outpatient department, the case of Dilchora referral hospital, Eastern Ethiopia: an institutional retrospective cross-sectional study

**DOI:** 10.1186/s40545-023-00539-y

**Published:** 2023-02-21

**Authors:** Tamiru Sahilu, Zenebe Kano

**Affiliations:** 1 Department of Pharmacy, College of Health Science, Assosa University, Assosa, Ethiopia; 2 School of Pharmacy, College of Medical and Health Science, Haramaya University, Dire Dawa, Ethiopia

**Keywords:** Antibiotics, Prescribing indicator, Prescribing practice, Urinary tract infections, Ethiopia

## Abstract

**Background:**

Inappropriate prescription of antibiotics is a global public health challenge. Widespread use, misuse, or inappropriate prescribing has resulted in unnecessary expenditure on drugs, raised risk of adverse reactions, the development of antimicrobial resistance, and increment in health care costs. There is a limited practice in rational prescribing of antibiotics in the management of Urinary tract infection (UTI) in Ethiopia.

**Objective:**

To assess antibiotic prescribing practice in the treatment of patients with UTI at outpatient department (OPD), Dilchora referral hospital, Eastern Ethiopia.

**Methods:**

A retrospective cross-sectional study was conducted from January 7 to March 14, 2021. Data were collected from 600 prescription papers using systematic random sampling method. World Health Organization’s standardized core prescribing indicators was used.

**Results:**

A total of 600 prescriptions containing antibiotics prescribed for patients with UTIs were observed during the study period. Of these, 415 (69.19%) were females and 210 (35%) were in the age group of 31–44 years. The number of generic drugs and antibiotics prescribed per encounter was 1.60 and 1.28, respectively. The percentage of antibiotics per prescription was found to be 27.83%. About 88.40% of antibiotics were prescribed by generic names. Fluoroquinolones were the most frequently prescribed class of drugs for the treatment of patients with UTIs.

**Conclusion:**

The prescribing practice of antibiotics in patients with UTIs was found to be good as the drugs were prescribed in generic name.

## Background

Irrational prescribing is a global problem. Bad prescribing habits lead to ineffective and unsafe treatment, exacerbation or prolongation of illness, distress and harm to the patient, and higher costs. Irrational prescribing patterns are perpetuated through patient pressure, and high-powered salesmanship by drug company representatives [1]. Among different irrationally prescribed, antibiotics are considered as most commonly prescribed and misused drugs [2, 3].

Antimicrobial drugs have been widely used in human medicine for more than 50 years either as prophylaxis or therapeutics, with tremendous benefits to human health. Unfortunately, widespread use, misuse, or inappropriate prescribing has resulted in the emergence of drug-resistant bacteria [4]. Antibiotic resistance is a global public health concern. Many studies have reported a positive relationship between antibiotic prescribing patterns and the level of antibiotic resistance [5]. The number of infections due to antibiotic-resistant bacteria is growing and outpacing the rate at which new classes of antibiotics are discovered and synthesized [6]. Antimicrobial resistance is also a barrier to public health efforts in the control of infectious diseases through specific disease control programs that rely on the use of antimicrobials as a strategy for control and prevention. Prudent prescribing and the use of antimicrobials helps to prevent the relentless increase in resistance. Currently, it is found that many microbes have become resistant to the most commonly available and effective first-line agents mainly due to inappropriate prescribing practices [7].

The consumption of antibiotics has increased worldwide with most of this occurring in low- and middle-income countries [8]. Antibiotic prescription varies significantly between countries as shown by 1 out of 2 hospitalized patients receiving antibiotics in Africa and Asia while 1 out of 3 patients in Europe receives antibiotics [9]. In many African countries, including Ethiopia, it is a common practice to prescribe multiple medications in a single prescription paper, which is referred to as polypharmacy, and the current study refers to using 5 and more medications in a single prescription paper.

UTI is an extremely common clinical problem that may involve the urethra, bladder, Uterus, and Kidney [10, 11]. It has been estimated that 150 million people were infected with UTIs per annum worldwide [10]. It affects all groups, but women are more susceptible than men due to short urethra, absence of prostate secretion, pregnancy, and easy contamination of the urinary tract with fecal flora [11, 12]. UTI is mostly caused by Gram-negative aerobic bacilli found in the GI tract. Included in this family are the *E. coli*, *Klebsiella*, *Enterobacter*, *Citrobacter*, *Proteus*, and *Serratia* species. Other common pathogens include *Staphylococcus epidermidis*, *Staphylococcus saprophyticus*, and *Enterococcus* species which presumably result in UTIs following colonization of the vagina or perianal skin [13].

Antibiotics are among the most frequently prescribed drugs and play a vital role in the treatment of infectious diseases [14]. Hence, they are the mainstay treatment for all UTIs. A variety of antibiotics are available and choices depend on many factors, including whether the infection is complicated or uncomplicated or primary or recurrent. Treatments should not necessarily be based on the actual bacterial count [15].

Fluoroquinolones are the most commonly used therapy for uncomplicated urinary tract infections [16]; however, the widespread use of fluoroquinolones for such a common infection raises concern regarding the possibility of accelerated development of resistance [17]. Recently, third-generation cephalosporin, especially ceftriaxone, was the most prescribed antibiotic for the treatment of patients with infectious diseases at most hospitals [18].

Antimicrobial resistance has increased rapidly and a major reason for this is the extensive use of drugs [19, 20]. Therefore, the emergency of antibiotic resistance has become a major issue of health care delivery; some of the prescribers-related factors that lead to increased resistance include lack of knowledge, inadequate diagnosis, incorrect drug selection, duration and route, prescription in response to patient pressure, financial gain and response to promotional pressure [21]. Many reports have indicated the presence of multidrug resistance in organisms causing UTIs [22].

World Health Organization (WHO) has designed standardized core prescribing and patient care indicators to evaluate the trends of drug use in outpatient settings of health facilities. Each core indicators have five components. The prescribing indicators include the degree of polypharmacy, the percentage of drugs prescribed with the generic name, the percentage of encounters with at least one antibiotic and injection, and the percentage of drugs prescribed from EDL (Essential Drug List). These indicators measure the performance of prescribers and dispensers in key areas concerning rational drug use with the appropriate prescription practice of antibiotics [23].

In Ethiopia, study in Shambu general hospital showed that from the prescribed dosage regimen of antibiotics 88.57% dose was reported as appropriate prescribed dose, 75.35% appropriate for frequency and 92.5% were appropriate for duration. The author concluded that there was irrational use of antibiotics during prescribing regarding dosage regimen appropriateness, drug interaction and contraindication [24]. The gap in Ethiopia seeks more attention as the practice of antibiotic prescribing for various infections is poor, most health facilities do not have their own guideline and do not adhere strictly to the national guideline.

This study helps to understand the possible gaps in antibiotic prescribing practice of the hospital and addressing them aggressively. Level of antibiotics prescribing practice was little known in the study area. Therefore, the study aimed to assess antibiotic prescribing patterns among patients with urinary tract infection at OPD of Dilchora referral hospital.

## Methods

### Study area and period

The study was conducted at the outpatient department pharmacy of Dilchora referral hospital (DRH). DRH is located in Diredawa town, Eastern Ethiopia. Dire Dawa is one of the two chartered cities in Ethiopia and is located 526 km from Addis Ababa in the East and 313 km to the west of Port Djibouti. DRH was established in 1952 E.C and is one of the oldest public hospitals found in the Eastern part of the country that runs under the Diredawa city administration. It is currently the only referral hospital in the city and serves nearly 1.5 million people. The hospital has more than 250 beds, 1600 staff members, and 22 care units. The hospital has medical, surgical, emergency, pediatrics, gynecology, ENT, and dermatology wards with their respective OPD. Of the listed wards, the OPD was the major source of patient encounters for this study. The OPD of the hospital will be an important platform for conducting the drug prescribing practice in UTIs. The study was conducted from Jan 7/2021 to March 14/2021.

### Study design

A hospital-based retrospective cross-sectional study was conducted to assess antibiotic prescribing practice in patients with UTI in DRH.

### Inclusion and exclusion criteria

#### Inclusion criteria:

- Prescription paper/encounter with patients aged ≥ 18 years of either sex, and
- Prescriptions containing at least one antibiotic.

#### Exclusion criteria:

- Prescription papers with illegible handwriting and containing only medical supplies

### Sample size determination

Based on the WHO guideline for assessing the rationality of drug use in OPD of healthcare settings, at least 600 prescribing encounters are considered for evaluation of prescribing practice if a single healthcare setting is selected [23]. Therefore, to imitate the guideline, 600 prescribing encounters were taken for the study. Within the past 6 months, about 12,600 prescriptions were found to be dispensed in the OPD.

### Sampling technique

Specific prescription papers were selected using a systematic random sampling method, using the prescription date as a reference for a sampling frame. The total number of prescription papers dispensed between July 1, 2020, and December 30, 2020, was 12,600; thus, dividing 12,600 by the number of the sample size “600” equals the sampling interval “21”. All prescriptions were arranged in ascending order of the date of the prescription. Then, every 21st prescription paper was selected.

### Study variables

#### Dependent variable

- Antibiotics prescribing practice in patients with UTI (Dosage regimen, and WHO indicator).

#### Independent variables

- Patient characteristics,
- Diagnosis-qualification of prescribers,
- STG (standard treatment guideline), facility specific/policy factor availability, and accessibility.

### Operational definitions

*Prescription completeness* All parameters that are indicated in the prescription paper have to be completed by the prescribers. These are patient information (patient full name, sex, age, weight, card number), treatment information (medicine generic name, strength, dosage form, dose, frequency, duration of treatment), and professionals’ information (prescriber’s full name, qualification, and signature, dispenser’s full name, qualification and signature, date of prescribing and dispensing.

*Prescription practice* The extent and profile of drug use, trends, quality of drugs, and compliance with regional, state, or national guidelines like standard treatment guidelines, usage of drugs from essential drug lists, and use of generic drugs (implies rational prescribing and dispensing).

*Prescribing error* Are error that occurs, as a result of a prescribing decision or prescription writing process and is classified as omission error related to the prescriber (including patient name, age, prescriber name, prescriber signature, the patient visited the department, and diagnosis), omission errors related to drugs (including route, dose, frequency, dosage form and quantity to supply) and commission errors (including wrong strength, wrong drug name not spelling, drug dosage form and drug–drug interaction).

### Data collection tools

The data collection tool was designed after reviewing relevant literature and from the WHO indicator, which was used to obtain information on antibiotic prescribing practices in UTIs. Data were collected from prescription paper and prescription registration book and prescribed with at least one antibiotic for UTI using: socio-demographic data, WHO indicators, any drug prescribed together, and the specific type of data necessary to measure the prescription paper was recorded for each patient encounter. The questionnaire was prepared in the English language. The data on prescriptions were collected by four well-trained data collectors using the data collecting format.

### Data collection procedures

Before commencing data collection, permission was received from the Dilchora referral hospital. The specific types of data necessary to measure the prescription paper were recorded for each patient encounter and entered directly into an excel sheet. The checklist was used for tracking data collected on prescription paper.

### Data processing and analysis

Data were entered into epi-data version 4.02.01 for cleaning purposes. The data were exported to SPSS version 24 for analysis. A descriptive cross-sectional analysis was carried out and results were presented by text, tables, and charts.

### Data quality assurance

A carefully designed data collection tool comprising variables to collect important data required to meet the stated objectives was used. The data collection tool was developed in English. Before the actual data collection process, a 5% pre-test was conducted on 10 patients' encounters (prescription paper) to evaluate the data collection tools. Four data collectors (two pharmacists with a B.Pharm degree and two clinical nurses with a BSc degree) were hired and one-day training on the data collection tool and general procedures were provided by the principal investigator. The data collection was supervised daily. All filled data collection checklists were reviewed for completeness and consistency on daily basis by the principal investigator.

### Ethical considerations

An ethical clearance letter was obtained from Harar Health Science College Research Ethics Review Committee and it was submitted to Dilchora referral hospital to obtain permission and cooperation for the study. Name of the patients on the prescription was not stated, data were kept secret and anonymous and it is only used for research purposes.

## Results

### Demographic data of the patients

During the 3-month study period, 600 prescriptions containing antibiotic indication for the treatment of UTI was reviewed. Demographic data of the patients (age and sex), as well as the date of prescription, were mentioned in all the prescriptions. Majority of the patients were females, 415 (69.19%). Most of the patients were in the age group of 31–44 (35%), followed by 45–64 (30%) (Table 1).

**Table 1:** Demographic characteristics of patients diagnosed with UTI in Dilchora hospital, Diredawa, 2021

Parameter	Category	Frequency	Percentage
Sex	Male	185	30.83%
	Female	415	69.19%
Age	20–30	120	20%
	31–44	210	35%
	45–64	180	30%
	≥ 65	90	15%

### Completeness of the prescription

According to the WHO guideline for a single healthcare setting, 600 outpatient prescription encounters were included in this study. Initially, the completeness of individual prescriptions was assessed before the prescribing indicator study. The date of prescription was written for about 550 (91.67%) prescriptions. In two-thirds of prescription papers, the patient’s name was written. Good prescribing practice was observed regarding sex, age, and medical registration number; whereas, poor recording practices were observed in weight (2.50%), diagnosis (4.67%), and address of the patients (5%) (Table 2).

**Table 2:** Completeness of patient-related information in prescriptions dispensed for patients diagnosed with UTI in DRH, 2021

Parameter	Category	Frequency	Percentage
Completeness of the prescription (*n* = 600) (superscriptions)	Used standard prescription paper	600	100%
	Date	550	91.67%
	Name of the patient	406	67.60%
	Sex	600	100%
	Age	600	100%
	Weight	15	2.50%
	Address	30	5%
	Medical record number	561	93.50%
	Diagnosis	30	4.67%

### Drug-related information on prescriptions dispensed

The name of the drug was written on all prescriptions. Majority of the prescriptions had the dose of the drug (87.33%), drug strength (87.33%), route (85.67%), frequency of administration (85%), and duration of treatment (73%). Poor prescribing practice was observed in the dosage form of the drug (18.50%) and the total quantity to be taken during treatment (35.34%) (Table 3).

**Table 3:** Drug-related information on prescriptions dispensed among patients diagnosed with UTI in Dilchora hospital, Diredawa, 2021

Parameter	Category	Frequency	Percentage
Drug-related information (Inscription)	Drug name	600	100%
	Drug strength	525	87.50%
	Dosage form	111	18.50%
	Route	514	85.67%
	Dose	524	87.33%
	Frequency	510	85%
	Quantity	212	35.34%
	Duration	438	73%

### Professionals related information

The third section of the prescription is about professionals’ information. The prevalence of written prescribers’ name and signature was found 258 (43%); whereas, the dispenser’s name and signature were found on only 68 (11.33%) prescriptions (Table 4).

**Table 4:** Prescriber and dispenser information on prescription dispensed for patients diagnosed with UTI in Dilchora hospital, Diredawa, 2021

Parameter	Category	Frequency	Percent
Professionals’ related information	Name and signature of the prescriber	258	43%
	Name and signature of the dispenser	68	11.33%

### Antibiotic prescribing practice

In this study, out of 968 medicines prescribed, 767 (79.24%) were found to be antibiotics. The most commonly prescribed antibiotic was fluoroquinolones accounting for 507 (66.10%), followed by penicillins 156 (20.34%), cephalosporins 56 (7.30%), and sulfonamides 48 (6.26%). Among the fluoroquinolones, the most frequently prescribed drug was ciprofloxacin 380 (74.95%) and norfloxacin 127 (25.05%). Whereas, amoxicillin–clavulanic acid 95 (60.90%) and amoxicillin 61 (39.10%) were the frequently prescribed antibiotics from class of penicillins (Table 5). Among others, omeprazole 80 (39.80%) and tramadol 30 (14.93%) were the most frequently prescribed drugs (Table 6).

**Table 5:** Antibiotics prescribed for patients with UTI in Dilchora hospital, Diredawa, 2021

Parameter	Category	Frequency	Percentage
Fluoroquinolones	Ciprofloxacin 500 mg tab	380	74.95%
	Norfloxacin 400 mg tab	127	25.05%
Penicillin	Amox-Clav 625 mg tab	95	60.90%
	Amoxicillin 500 mg caps	61	39.10%
Cephalosporin	Cephalexin 500 mg caps	56	7.30%
Sulfonamides	Co-trimoxazole 960 mg tab	48	6.26%

**Table 6:** Other drugs prescribed with antibiotics for patients with UTI in Dilchora referral hospital, Diredawa, 2021

Drugs	Frequency	Percentage
Tramadol 50 mg	30	14.93%
Diclofenac 50 mg	14	6.97%
Glibenclamide 5 mg	12	5.97%
Metformin 500 mg	15	7.46%
Omeprazole 20 mg	80	39.80%
Metronidazole 250 mg	15	7.46%
Cimetidine 200 mg/5 ml	15	7.46%
Ibuprofen 400 mg	20	9.95%
Total	201	100%

### Number of antibiotics per prescription

Of 600 prescriptions analyzed, 76 (12.67%) prescriptions contained more than 2 antibiotics, 16 (2.67%) prescriptions contained three antibiotics and 524 (87.33%) prescriptions contained one antibiotic.

### Antibiotic prescribing errors

The analysis of the antibiotic prescribing errors showed that a total of 171 (28.52%) prescribing errors were detected. All of the errors were identified to be omission errors. The commonly omitted information was dosage form 61 (10.17%), quantities of medications 49 (8.16%), and duration of therapy 20 (3.33%) (Table 7).

**Table 7:** Antibiotics prescribing errors observed in patients with UTI in Dilchora hospital, Diredawa, 2021

Parameter	Category	Frequency	Percentage
Antibiotics prescribing errors observed	Strength of drugs	9	1.51%
	Dosage form	61	10.17%
	Quantities of medications	49	8.17%
	Route of administrations	12	2%
	Frequency of administration	11	1.83%
	Duration of therapy	20	3.33%
	Strength of drugs	9	1.51%
Total error		171	28.52%

### Prescribing pattern indicators with respect to WHO references

The average number of antibiotics per prescription and percentage of encounters with an injection were 1.28 and 15.50%, respectively. The majority of drugs (90.19%) were prescribed with generic names. All drugs were prescribed from the Essential Drug List (Table 8). Percentage of encounters with antibiotics indicated that more than one antibiotic was contained per prescription.

**Table 8:** Prescribing pattern indicators with respect to WHO references

Parameter	Category	Values		
		Total drugs	Antibiotics	WHO reference
Who Indicators	The average number of drugs per prescription	968/600 = 1.6	767/600 = 1.278	1.60–1.99
	Percentage of encounters with an injection prescribed	93/600 = 15.5%	–	13.4–24.1
	Percentage of encounters with antibiotics	767/600 = 127.83%	167/600 = 27.83%	20–26.80
	Percentage of drugs prescribed by generic name	873/968 = 90.19%	678/767 = 88.40%	100
	Percentage of drugs from Essential Drug List/EDL	100%	100%	100

## Discussion

The writing of correct and complete drug prescriptions is an important component of the scientific process. Rational drug prescription avoids many adverse drug reactions and complications, which arise from inappropriate prescribing of drugs. Omitting data on prescribed drugs could lead to numerous problems including under or over-treatment of the patients [25]. In this study, the prescribing antibiotic pattern analysis showed that a total of 161 (26.83%) prescribing errors. All of the detected errors were identified to be omission errors. This might be due lack of responsibility and patient overload.

In the present study, about 88.40% of antibiotics were prescribed by generic names. This is encouraging, as it is in line with the WHO recommendation. Our result was lower than a study done in Hawassa, Addis Ababa, and Wolayita Sodo in which generic prescribing constituted 98.14%, 98.70%, and 94%, respectively [26–28]; whereas, our finding showed greater prevalence than the study done in Kenya, 62.50% of drugs were prescribed by generic names [29]. However, the current study contradicted the study conducted in the US which showed that prescribers preferred brand medicines over generic and the percentage of drugs prescribed by brand name was 80% [30]. This might be due to differences in the countries’ medication procurement policy in which our country policy promotes procurement by generic name.

The number of drugs prescribed per encounter should be as low as possible to minimize the risk of drug interaction, development of drug resistance, and medication cost. In this study, the number of generic drugs and antibiotics prescribed per encounter was 1.6 and 1.28, respectively. This result is in line with WHO recommendation which states an average of not more than 1.99 drugs should be written per prescription. On the other hand, the present result is different from a previous study done in a teaching hospital, in western Nepal, 1.76 antibiotics per prescription [31]. This might be due to the applied antibiotics prescription policy, involvement of pharmacists in the multidisciplinary team, and strict follow-up by the hospital management.

The percentage of antibiotics per prescription was found to be 27.83%, which is greater than the ideal value recommended by WHO (20–26.80%) [32]. This finding was lower than previous study conducted in Gondar, Addis Ababa, and southern Ethiopia, in which antibiotics constitute 29.30%, 38%, and 58.10%, respectively [8, 27, 28]. This might be due to the prescribing habit and the difference in the prevalence of infectious diseases in the area.

Fluoroquinolones were the most frequently prescribed class of drugs for the treatment of UTIs in the present study. Among the fluoroquinolones, the most frequently prescribed drug was ciprofloxacin 380 (74.95%), followed by norfloxacin 127 (25.05%). The possible reason is that these agents are used as first-line drugs for the treatment of UTIs in the country. The present observation is different from the previous study conducted in Malaysia, in which penicillin was the most frequently prescribed drug, followed by co-trimoxazole [33]. On the contrary, Chowta [34] reported that cephalosporin was the most commonly used drug for the treatment of UTIs. Our finding was inconsistent with a similar study conducted in Addis Ababa and Dessie, in which penicillin (51.90%) were the most commonly prescribed groups of antibiotics followed by Fluoroquinolones (18.30%) [19, 27]. This might be due to regional variation in bacterial susceptibility/resistance, prescribing habits, and the difference in the prevalence of infectious diseases in different region.

The percentage of encounters with an injection prescribed was calculated to measure the overall level of use of commonly overused and costly forms of drug therapy. The percentage of drugs prescribed by injection in this study was 15.50% of the total drugs, which is found to be within the acceptable range recommended by WHO. However, there are no injectable antibiotics reported in this study. This is due to the study being done in the outpatient department and antibiotics used in this department for the treatment of UTIs were in the oral dosage form.

In this study, the percentage of antibiotics prescribed from the EDL was 100%, which was in line with the ideal value set by WHO. This result of the current finding showed good prescribing practice and it could be due to a strict follow-up by the hospital management and/or it could be due to the pharmaceutical procurement policy of the country, which is based on the EDL of the country, and this limits prescribers not to prescribe drugs out of the list because only drugs from the EDL were available in the health care facility.

*Limitation of the study* The study was conducted only in the OPD and thus does not include antibiotics utilization in inpatient departments and thus may not correctly indicate the entire antibiotics prescribing pattern in the hospital. The data were also collected only by reviewing the prescription papers and registry books, which does not include interviews with prescribers and/or patients and thus does not assess factors contributing to the current practice. The study also does not include a review of the medical chart of patients to further evaluate if the prescriptions were rational and appropriate to the current diagnosis; except for a few diagnoses written on the prescription.

## Conclusion

The prescribing patterns of antibiotics among patients with UTI were found to be good, as the drugs were prescribed from the national treatment guideline and in their generic name. Moreover, the majority of drugs were prescribed as monotherapy. However, some important information was frequently missing from the prescription such as the physician's signature, dosage form, quantities of medications, and route of administration. Omission of frequency of administration, duration of therapy, and strength of drugs were the least prevalent errors observed. Overall, the antibiotics prescribed for patients with UTIs in the OPD were fluoroquinolones particularly, ciprofloxacin, followed by norfloxacin.

## Data Availability

The data sets generated during and/or analyzed during the current study are available from the corresponding authors upon reasonable request.

## Abbreviations

ADR: Adverse drug reactions
CDC: Center for Disease Control
DRH: Dilchora referral hospital
DUE: Drug use evaluations
DUR: Drug utilization review
ECDC: European Center for Disease Control
EDL: Essential Drug List
EMA: European Medicine Agency
ETB: Ethiopian Birr
LMIC: Low- and middle-income country
OPD: Outpatient department
SPSS: Statistical Package for Social Science
UTI: Urinary tract infections
WHO: World Health Organization
STG: Standard treatment guideline
